# The relation between the blood glucose level and the FDG uptake of tissues at normal PET examinations

**DOI:** 10.1186/2191-219X-3-50

**Published:** 2013-07-06

**Authors:** Henry Lindholm, Fredrik Brolin, Cathrine Jonsson, Hans Jacobsson

**Affiliations:** 1 Department of Radiology, Karolinska University Hospital Solna, Stockholm, SE 171 76, Sweden; 2 Department of Hospital Physics, Karolinska University Hospital, Stockholm, SE 171 76, Sweden

**Keywords:** Hyperglycaemia, FDG/PET, Normal tissue, SUV, Tracer distribution

## Abstract

**Background:**

The influence of the blood glucose level on the tracer uptake of normal tissues at [^18^F]-2-fluoro-2-deoxy-d-glucose (FDG) positron emission tomography (PET) was retrospectively studied in examinations in clinical patients.

**Methods:**

Five hundred examinations were evaluated in retrospect. The inclusion criteria were studies with a normal or near-normal FDG distribution. Patients who had been subjected to chemotherapy (including GSF treatment) or radiotherapy <4 weeks prior to the examination were excluded; we cannot exclude, however, that in a very few patients the available information might have been incomplete. Otherwise, patients were included regardless of concurrent diseases and/or therapy. In one evaluation, the mean standardized uptake value of the liver, spleen, lungs, peripheral blood, selected muscles and bone marrow of all 500 individuals was correlated to the blood glucose level. In another evaluation, a subgroup of 62 patients with increased blood glucose levels (≥7.0 mmol/l) was compared with another subgroup of 62 patients paired with regard to age and gender with blood glucose levels within normal range (≤6.0 mmol/l).

**Results:**

There was a weak positive correlation between the blood glucose level and the muscular uptake of FDG, while there was no correlation with the tracer uptake of the liver, spleen, lungs, peripheral blood or bone marrow. The patient group with increased blood glucose levels showed a slightly, but significantly, higher muscular FDG uptake compared with the matched subgroup of patients with normal blood glucose levels. When comparing the other assessed tissues/organs, there were no differences between these two patient groups.

**Conclusions:**

The effect of hyperglycaemia at FDG PET on the studied normal tissues is restricted to a slightly increased muscular uptake. The effect of the blood glucose level on the blood activity at the time of examination is negligible.

## Background

When clinical [^18^F]-2-fluoro-2-deoxy-d-glucose (FDG) positron emission tomography (PET) was established in the early 1990s, there were reports that hyperglycaemia impaired the tumour uptake of FDG because of competition with endogenous blood glucose (B-glucose) [1-7]. This led to recommendations in the European [8] and American [9] guidelines to measure the B-glucose concentration prior to FDG PET and reschedule patients with values exceeding a certain level. While initially adhering to the recommendations, we encountered patients with elevated B-glucose levels who were difficult to reschedule which is why they were examined despite the evaluation. The reasons for doing so included an urgent need for the examination, a long travel distance for the patient and, in most cases, the patient’s unwillingness to be rescheduled. At visual evaluation the image quality of these examinations did not differ from the image quality obtained in clinical patients with a normal B-glucose. This observation together with a number of subsequent reports questioning the effect of hyperglycaemia on the distribution of FDG has increasingly caused us to accept such patients for clinical examination.

There are currently a number of reports on the effect of hyperglycaemia and/or diabetes mellitus on the image quality at FDG PET with somewhat opposing findings [10]. Thus far, all the studies have focused on the tracer uptake by the pathological lesion, and there has been very little focus on the uptake by surrounding normal tissues, even though the uptake by surrounding tissues comprises the background activity from which the lesion should be discerned. In this study we retrospectively assessed the standardized uptake values (SUVs) of a number of normal tissues/organs in clinical patients with normal or near-normal FDG distributions and analysed the correlation with B-glucose. The radiotracer distribution in normal tissues/organs was also compared between two subgroups of patients matched with regard to age and gender; one subgroup had increased B-glucose levels, and the other subgroup had B-glucose levels within normal range.

## Methods

### Study design

FDG PET/computed tomography (CT) studies with a normal or near-normal activity distribution were included. All reports from clinical examinations were retrospectively evaluated in a ‘backwards’ order. Between September 2012 and October 2009, 500 examinations fulfilled our inclusion criteria. Patients who had been subjected to chemotherapy (including GSF treatment) or radiotherapy <4 weeks prior to the examination were excluded; we cannot exclude, however, that in a very few patients the available information might have been incomplete. Otherwise, patients were included irrespective of concurrent diseases and/or therapy. Examples of near-normal activity distributions were small, probably benign lung lesions or radiation portals with an FDG uptake not exceeding normal soft tissue activity, apparent reactive normal-sized lymph nodes with a moderately increased uptake, and a weak uptake in surgical scars and accumulation in minor, presumably benign skin lesions. Patients with a very small FDG extravasation at the injection site were studied, while individuals with larger extravasations were excluded. Similarly, only patients with a minute brown adipose tissue uptake were included. Patients with a visually apparent generally increased muscular uptake as previously studied by us were not included [11]. No patient with evidence of active malignancy was studied.

In the first evaluation, the correlation between the blood glucose level and SUVmean of various normal tissues was assessed in all 500 patients. In the second evaluation, patients with a B-glucose ≥7.0 mmol/l (*increased*) were paired with regard to age and gender with patients with a B-glucose ≤6.0 mmol/l (*normal*). Of 75 patients with an increased B-glucose, 62 patients could be matched with normals and were studied.

### Patients

In the first evaluation, all 500 patients were studied. The mean age was 58 years (range 11 to 89). There were 248 males and 252 females. Mean B-glucose was 6.0 mmol/l (±SD 1.6). The second (paired) evaluation comprised 62 + 62 patients with a mean age of 65 years (range 25 to 80), including 33 males and 29 females in each group. In the patients with an increased B-glucose, the mean B-glucose concentration was 9.0 mmol/l (±SD 2.0) and the mean body mass index (BMI) was 26.5 kg/m^2^ (±SD 4.9). Of these patients, 26 were known diabetics, 13 of whom were on insulin treatment. In the controls, the mean B-glucose concentration was 5.2 mmol/l (±SD 0.6) and the mean BMI was 24.3 kg/m^2^ (±SD 3.3). One of these patients was a known diabetic on insulin treatment. There was a strongly significant difference of the B-glucose between the two subgroups (*p* < 0.0001). This retrospective investigation was approved by the regional ethics research committee (Regionala etikprövningsnämnden i Stockholm, Karolinska Institutet, SE-171 77 Stockholm, Sweden; unique identifying numbers 2009/1491-31/2 and 2012/1434-32).

### PET/CT examinations

B-glucose was measured immediately prior to administration of FDG using the same glucometer, HemoCue® Glucose 201^+^ (Hemocue AB, Ängelholm, Sweden).

A Biograph 64 TruePoint TrueV PET/CT scanner (Siemens Medical Solutions, Erlangen, Germany) was used. The examination was performed according to the European guidelines, although patients with increased B-glucose were not rescheduled [8]. FDG (4 MBq/kg body weight) was administered intravenously (i.v.). The examination was performed approximately 1 h thereafter. It usually included the mid-skull to the proximal thigh. Prior to examination with i.v. contrast medium, a low-dose CT was performed for attenuation and scatter correction. Directly thereafter, the PET examination was done, followed by a diagnostic (full-current) CT with or without i.v. contrast medium. At examinations without i.v. contrast medium, the full-dose CT was used for attenuation and scatter correction.

The diagnostic CT examinations were performed with a tube tension of 120 kV, a pitch of 0.8, a slice thickness of 1.2 mm and a rotation speed of 0.5 s. The current was set to 160 mAs (reference), and dose modulation (CARE Dose4D) was applied. In the examinations done only for photon attenuation and scattering correction, CT was performed with a tube tension of 120 kV, a pitch of 0.8, a slice thickness of 1.2 mm, a rotation speed of 0.5 s and a current of 50 mAs. CT acquisitions were always done with the breath-holding technique at a mean inspiratory level.

There was a mean of 61 min (range 50 to 70 min) between tracer administration and PET registration. 3D PET acquisition was done for 3 min at each bed position during normal tidal breathing. The PET data were subsequently reconstructed with the manufacturer’s 2D-OSEM algorithm (four iterations and eight subsets) using a 5-mm post-reconstruction Gaussian filter. The image matrix size was 168 × 168 with a slice thickness of 5 mm. In addition to attenuation and scatter correction, all data were corrected for dead time and random coincidences.

### Image analysis

Activity quantification was done in the PET images using Siemens syngo MultiModality Workplace (syngo MMWP, VE36A). Volumes of interest (VOIs) of different sizes and shapes adapted to the various organs/tissues were drawn manually, avoiding margins.

The definition in the images of the various VOIs was done by combining the information from both the CT and PET acquisitions. SUVmean of the liver was calculated by averaging the values of VOIs with a volume of 20 to 50 cm^3^ allocated at the centre of the right and left liver lobes, respectively. SUVmean of the spleen was similarly averaged from three elliptical VOIs with a volume of 4 to 8 cm^3^ in various portions of the organ. Muscular SUVmean was calculated by averaging the values of elliptical VOIs allocated in the right and left shoulder muscles (25 to 50 cm^3^), in both psoas muscles (5 to 10 cm^3^) and in the right and left gluteal muscles (25 to 50 cm^3^), respectively. SUVmean of the lungs was calculated by averaging the values of parasagittal, ‘flat’ VOIs with a volume of 25 to 50 cm^3^ in each lung, covering both the upper and lower lobes. Blood SUVmean was assessed by averaging the activity of five VOIs with a volume of 0.8 to 2 cm^3^ allocated in different portions of the lumen of the large mediastinal vessels. As they could not be discerned from the diffuse mediastinal FDG activity in most patients, allocation of these VOIs was done using the CT images. In cases of increased activity of the vessel wall, this was avoided. Bone marrow SUVmean was calculated by averaging the values of elliptical VOIs with a volume of 2 to 5 cm^3^ allocated in both iliac crests and spherical VOIs with a volume of 1 to 1.5 cm^3^ in each lumbar vertebral body. If a vertebra was not possible to evaluate because of a compression or extensive spondylosis, for example, it was excluded. In a few studies, one or two of the lower dorsal vertebrae were included instead.

### Statistical methods

The distribution of the data showed that the correlation between B-glucose and SUVmean of the various organs/tissues could be analysed by Pearson’s correlation coefficient, applicable for linear associations. SUVmean of the muscles showed a skewed distribution (>1), which is why a reciprocal transformation was carried out prior to the analysis. Otherwise, such corrections were not done. A correlation coefficient of 0 to 0.25 indicates *little or no* relationship. Correlation coefficients between 0.25 and 0.50 indicate a *fair degree* of relationship, those between 0.50 and 0.75 indicate a *moderate to good* relationship and those >0.75 indicate a *very good to excellent* relationship [12]. The coefficient of determination (*R*^2^) indicates the proportion of the total variation in SUVmean that is explained by the variable studied, i.e. B-glucose.

The distribution of the data allowed for the use of Student’s two-tailed *t* test for independent samples for assessing the difference in SUVmean between the patients with increased B-glucose and the patients with normal B-glucose. The Mann–Whitney *U* test was applied to compare the B-glucose level of the two subgroups as the variable was not normally distributed. *P* < 0.05 was considered statistically significant.

## Results

In the analysis of the entire patient group, the skeletal muscles were distinct from the other organs/tissues (Table 1). The correlation coefficient (0.24) for skeletal muscles indicated an almost fair relationship with B-glucose, corresponding to an *R*^2^ of 0.06. The correlation coefficients for the other organs/tissues were much lower (0.01 to 0.14), although a significant difference from zero indicated a certain correlation for the liver, spleen and blood. The *R*^2^ for the organs/tissues other than the skeletal muscles was very low (0.00 to 0.02).

**Table 1:** Correlation between blood glucose concentration and mean SUVmean of various normal tissues/organs at FDG PET examination

	**Liver**	**Spleen**	**Lungs**	**Blood**	**Muscles**	**Bone marrow**
Correlation coefficient	0.14	0.10	0.01	0.12	0.24	0.06
Significance (*p*)	0.001 ≤ *p* < 0.01	0.01 ≤ *p* < 0.05	ns	0.001 ≤ *p* < 0.01	<0.001	ns
*R*^2^	0.019	0.010	0.000	0.014	0.055	0.004

*ns*, not significant.

In comparing the patients with normal B-glucose values with the patients with increased values, the latter group showed a higher muscular FDG uptake (Table 2). The difference was very small but nevertheless significant. Otherwise, there were no significant differences between these two patient groups.

**Table 2:** Mean SUVmean (±SD) of various normal organs/tissues in clinical patients at FDG PET examination

	**Liver**	**Spleen**	**Lungs**	**Blood**	**Muscles**	**Bone marrow**
Increased B-glucose	2.5 (0.45)	1.8 (0.38)	0.4 (0.13)	1.6 (0.39)	0.9 (0.21)	1.5 (0.37)
Significance (*p*)	ns	ns	ns	ns	0.001 ≤ *p* < 0.01	ns
Normal B-glucose	2.4 (0.37)	1.7 (0.28)	0.4 (0.12)	1.6 (0.29)	0.8 (0.18)	1.4 (0.30)

Sixty-two patients with increased blood glucose concentrations (≥7.0 mmol/l) were compared with 62 patients matched with regard to age and gender, with normal blood glucose (≤6.0 mmol/l) levels. *ns*, not significant.

## Discussion

Normal phenomena should ideally be studied in normal individuals. As this is not possible when a large number of individuals must be studied, clinical patients who are considered normal have to serve as subjects. Since the FDG distribution seems to be a zero-sum game [11], only patients with normal PET findings should ideally have been studied. This turned out to be difficult since few clinical patients show a completely normal FDG distribution. Consequently, examinations showing a minor uptake, which is assumed to have a negligible effect on the FDG distribution, were included. The shortcomings of basing the study on clinical patients should be well compensated for the large number of observations.

SUV assessments are strongly influenced by the partial volume effect, which in small lesions will lead to an underestimation of the uptake. In the current PET system, a VOI corresponding to a sphere with an approximate diameter of ≥2 cm (4.2 cm^3^) is necessary for an adequate recovery of the uptake. This should be the case in the assessments of the liver, lungs, muscles, iliac crests and, in most patients, the spleen, while in many patients the size of the mediastinal vessels and the vertebral bodies is not sufficient. However, this fact should hardly affect our findings because of the comparative nature of the study and, not least, the large number of observations.

The cut-off values used for defining the two patient groups in the second part of the study were chosen to distinguish clearly between the two groups in order to allow for an optimal comparison of the effects of B-glucose. They were not related to any values regarding the rescheduling of patients for examination.

The current study was inspired by a recent report that a high serum glucose concentration does not predict poor image quality in FDG PET/CT, which confirmed a growing impression of ours [13]. A reduction in the FDG uptake of the brain at increased B-glucose values has been convincingly demonstrated [2,14,15]. There is also a well-known opposite effect on the myocardial uptake of FDG [16]. With the exception of these two tissues, previous studies of the effects of B-glucose and/or diabetes on the accumulation of FDG in normal tissues are not consistent. All such studies have also been performed in much smaller patient cohorts than in the present investigation. An increased background activity with a consequent lowered image quality was reported in a series of patients with pancreatic cancer who were subjected to *chronic* hyperglycaemia [6]. Another study of patients with the same condition showed a reduced sensitivity of various malignancies but not of inflammatory lesions [10]. After *glucose loading*, an increased background activity leading to a lowered image quality was reported in a series of patients with colorectal tumours [17], as well as a somewhat more muscular FDG uptake in a number of patients with head and neck cancer [3]. In contrast to these findings, there are two studies reporting no effects on the soft tissue background activity with *chronically* increased B-glucose in tumour patients [18,19]. In another clinical study, chronic hyperglycaemia did not affect the FDG uptake in the liver or muscles, while the brain uptake decreased [14]. A similar finding was reported in rats after a *glucose load*[15]. Another study in rats with *experimentally induced* hyperglycaemia showed higher FDG activity in the blood compared to controls and a much lower brain uptake, while the muscular activity was insignificantly affected [2]. In summary, in cases of an effect exerted by increased B-glucose, a *higher* uptake by normal tissues outside the brain is reported. It also seems that the FDG uptake of normal tissues is affected more by *acute* (experimentally induced) hyperglycaemia than by chronic hyperglycaemia [14].

The key finding of this study is the higher FDG uptake of the muscles with increased B-glucose, which was found in both evaluations. The effect is weak but is distinct from the effect on other tissues where the correlations can, in principle, be disregarded and considered to be caused by the very large number of observations. The described effect is in line with previous reports since the patients with increased B-glucose in this study were not subjected to induced hyperglycaemia and in most cases must be considered to be suffering from chronic hyperglycaemia. The conclusion is that the effect of increased B-glucose on the FDG uptake of various normal tissues can be ignored in clinical examinations.

The tendency toward a correlation between B-glucose and the FDG activity of the muscles is a clear finding and is line with most previous reports. It is, however, not consistent with the theory of a pure competition between FDG and endogenous B-glucose giving rise to a lower tracer uptake by various ‘indifferent’ tissues. Such a straightforward mechanism is also contradicted by the negligible effect of B-glucose on the blood activity, which is a fundamental finding of the study. A competition between FDG and endogenous B-glucose would give rise to an increased blood activity with an increased B-glucose. The cellular glucose uptake is a complex mechanism influenced by several insulin-dependent as well as non-insulin-dependent factors, but a thorough analysis of this mechanism is beyond the scope of this work. Like glucose, the transfer of FDG into the cells is mediated by the glucose transporters 1 to 7 (Glut1 to Glut7) and the sodium-glucose-linked transporters 1 to 2 [10]. Most glucose transporters are expressed in a tissue-specific manner. Interestingly, there is a large expression of Glut4 transporters in fat, skeletal muscle and heart [20,21], and thus, the latter two tissues show an increased FDG activity with increased B-glucose.

## Conclusions

The effect of hyperglycaemia at FDG PET on the studied normal tissues is restricted to a slightly increased muscular uptake. The effect of the blood glucose level on the blood activity at the time of examination is negligible.

## Competing interests

The authors declare that they have no competing interests.

## Authors’ contributions

HL carried out many evaluations and participated in the study design, interpretation of the data and drafting of the manuscript. FB participated in the study design and was responsible for the examinations. CJ participated in the study design, interpretation of the data and drafting of the manuscript. HJ conceived the study, carried out many evaluations and participated in the interpretation of the data and drafting of the manuscript. All authors read and approved the final manuscript.
